# Clinical and genetic characterization of a cohort of 97 CLN6 patients tested at a single center

**DOI:** 10.1186/s13023-022-02288-8

**Published:** 2022-05-03

**Authors:** Corina-Marcela Rus, Thomas Weissensteiner, Catarina Pereira, Iuliana Susnea, Bright D. Danquah, Galina Morales Torres, Maria Eugenia Rocha, Claudia Cozma, Deepa Saravanakumar, Sumanth Mannepalli, Krishna K. Kandaswamy, Sebastiano Di Bucchianico, Ralf Zimmermann, Arndt Rolfs, Peter Bauer, Christian Beetz

**Affiliations:** 1 Centogene GmbH, Am Strande 7, 18057 Rostock, Germany; 2 Institute of Chemistry, University of Rostock, Dr.-Lorenz-Weg 2, 18059 Rostock, Germany; 3 Helmholtz Zentrum München, Ingolstädter Landstraße 1, 85764 Neuherberg, Germany; 4 Arcensus GmbH, Goethestrasse 20, 18055 Rostock, Germany; 5 Department of Medicine, Clinic III, Hematology, Oncology, Palliative Medicine, University of Rostock, Rostock, Germany

**Keywords:** Rare disease, Lysosomal storage disorder, Batten disease, Neuronal ceroid lipofuscinoses, *CLN6*, New variant, Genotype, Phenotype

## Abstract

**Background:**

Ceroid lipofuscinoses neuronal 6 (CLN6) disease belongs to the neuronal ceroid lipofuscinoses (NCLs), complex and genetically heterogeneous disorders with wide geographical and phenotypic variation. The first clinical signs usually appear between 18 months and 8 years, but examples of later-onset have also been reported. Common manifestations include ataxia, seizures, vision impairment, and developmental regression. Because these are shared by other neurological diseases, identification of *CLN6* genetic variants is imperative for early diagnosis.

**Results:**

We present one of the largest cohorts to date of genetically diagnosed CLN6 patients screened at a single center. In total 97 subjects, originating from 20 countries were screened between 2010 and 2020. They comprised 86 late-infantile, eight juvenile, and three adult-onset cases (two patients with Kufs disease type A, and one with teenage progressive myoclonic epilepsy). The male to female ratio was 1.06: 1.00. The age at referral was between six months and 33 years. The time from disease onset to referral ranged from less than 1 month to 8.3 years. The clinical phenotype consisted of a combination of symptoms, as reported before. We characterized a total of 45 distinct variants defining 45 distinct genotypes. Twenty-four were novel variants, some with distinct geographic associations. Remarkably, c.257A > G (p.H86R) was present in five out of 23 unrelated Egyptian individuals but in no patients from other countries. The most common genotype was homozygosity for the c.794_796del in-frame deletion. It was present in about one-third of CLN6 patients (28 unrelated cases, and 2 familial cases), all with late-infantile onset. Variants with a high likelihood of causing loss of CLN6 function were found in 21% of cases and made up 33% of all distinct variants. Forty-four percent of variants were classified as pathogenic or likely pathogenic.

**Conclusions:**

Our study significantly expands the number of published clinical cases and the mutational spectrum of disease-associated *CLN6* variants, especially for the Middle Eastern and North African regions. We confirm previous observations regarding the most prevalent symptoms and recommend including *CLN6* in the genetic diagnosis of patients presenting with early-onset abnormalities of the nervous system, musculoskeletal system, and eye.

## Background

Neuronal ceroid lipofuscinoses (NCLs) are a genetically heterogeneous group of inherited lysosomal storage disorders [1, 2]. Together, they constitute the most prevalent class of rare childhood-onset neurodegenerative diseases [3–5]. The estimated total incidence of all NCLs ranges from 0.01 to 9 per 100 000 live births [6–8] but varies between countries and geographical regions [5]. NCLs are characterized by an accumulation of intracellular auto-fluorescent storage material (ceroid) and neurodegeneration [9]. The clinical spectrum consists of a combination of symptoms including intellectual and motor deterioration, visual impairment, seizures, psycho-motor decline, and loss of neurons [2]. The order in which these symptoms appear differs between the disease subtypes, but the outcome is always fatal. NCLs have been subclassified according to age at onset and clinical features into congenital (CNCL), infantile (INCL), late-infantile (LINCL), juvenile (JNCL), and adult (ANCL) neuronal ceroid lipofuscinoses. Thirteen genes with NCL associated variants have been established to date, named *CLN1-CLN8* and *CLN10-CLN14* [10, 11]. Additionally, a new subtype of NCL (CLN15) has been proposed [12]. The general pattern of inheritance is autosomal recessive, except for ANCL which can be either autosomal recessive or dominant [13]. Genetic heterogeneity and overlapping clinical features make the diagnosis of NCL disease challenging.

Ceroid lipofuscinosis neuronal protein 6, encoded by the *CLN6* gene, forms complexes with other proteins which act as key-regulators of vesicular sorting and trafficking. Defects can have a variety of consequences, from diminished lysosomal function to impaired neurotransmitter secretion and neurite outgrowth [14–16]. The worldwide incidence of CLN6 disease is currently not accurately known. The classical clinical subtypes are late-infantile and juvenile (OMIM# 601780). In addition, atypical phenotypes such as Kufs disease type A (OMIM# 204300), with or without teenage progressive myoclonic epilepsy, have been reported [17]. The major clinical manifestations of classical-onset CLN6 are similar to that of other NCLs and premature death typically occurs between five and 12 years of age [18]. Recent research has improved knowledge of the pathogenic mechanisms but therapy to delay disease progression exists so far only for patients with defects in *CLN2* [19]. However, *CLN6* gene therapy has shown encouraging results in mice and primates [20], and is currently being trialed in humans (NCT02725580) [20–22].

**Fig. 1:**
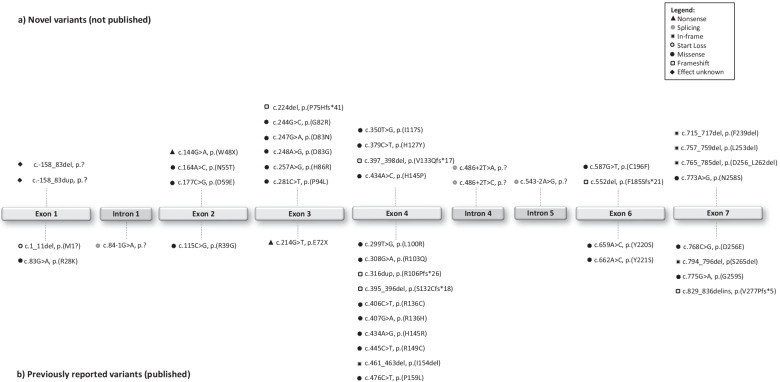
Schematic representation of the variant type and exon–intron distribution of the variants in CLN6. Each symbol represents the corresponding variant type (see legend). Novel variants identified in this study are shown above the grey boxes (**a**) and previously reported variants from this study are presented below the grey boxes (**b**). The majority of variants are missense variants. Most of the novel reported variants are located in exons 3, 4, and 7

Here, we present an analysis of a large cohort of patients (n = 97) who were referred to us for molecular genetic testing and diagnosed with disease-associated *CLN6* variants (Fig. 1). Our aims were a better understanding of the diversity of clinical symptoms and the characterization of *CLN6* variants, especially in geographical regions which were underrepresented in public datasets.

## Results

### Demographic and clinical characteristics of the cohort

At the time of writing, CentoMD® 5.8 [23] stored curated data for 97 individuals genetically diagnosed with CLN6 disease. These included 85 (87%) unrelated individuals and 12 (13%) familial cases. The familial cases were six sibling pairs with late-infantile disease and variable age of referral. Three sibling pairs were from Egypt (North Africa), two from Lebanon, and one from Saudi Arabia (Middle East). The following analysis was performed without accounting for family membership, except when noted and implicitly when calculating the frequency of distinct alleles in the cohort.

Patients originated from 20 countries, grouped into six geographical regions. Most cases were from the Middle East (59%) and North Africa (32%). Within these regions, respectively, Saudi Arabia (22%) and Egypt (27%) were the countries that contributed the greatest number of cases in the total cohort (Table 1). The ratio of males (n = 47) to females (n = 44) was nearly equal. According to categorical disease subtype information, 86 (89%) of patients had late-infantile, 8 (8%) juvenile, and 3 (3%) adult-onset disease [24]. The adult-onset cases comprised two Kufs disease type A, and one teenage progressive myoclonic epilepsy.

**Table 1:** *CLN6* gene variants and associated patient characteristics; entries are in bold for variants that have not previously been described

cDNA	Protein	Predicted effect	Clinical significance	Evidence (ACMG)	Disease subtype	Age(s) of onset [years]^†^	No. of times observed (sibling cases)	Patient origin
c.1_11del	p.M1?	LoF	VUS	PM2_P, PM2_P, PVS1_P	Late-infantile		1	Saudi Arabia
c.83G > A	p.R28K	Missense	VUS	PP3, PM2_P, PM3	Juvenile	2, 8.5	4	Oman (3), Saudi Arabia
c.84-1G > A	p.?	LoF	LP	PVS1_S, PM2_P, PM3_P	Juvenile		1	Sri Lanka
c.115C > G	p.R39G	Missense	VUS	PM2_P	Juvenile		1	Lebanon
**c.144G > A**	p.W48*	**LoF**	**LP**	**PVS1, PM2_P**	**Late-infantile**		**1**	**Jordan**
**c.-158_83del**	**p.?**	**LoF**	**LP**	**PVS1_S, PM2_P, PM3_P**	**Late-infantile**		**1**	**United Arab Emirates**
**c.-158_83dup**	**p.?**	**LoF**	**VUS**	**PM2_P, PVS1_S**	**Late-infantile**		**1**	**Saudi Arabia**
**c.164A > C**	**p.N55T**	**Missense**	**VUS**	**PM2_P, PP3**	**Juvenile**	**13**	**1**	**Egypt**
**c.177C > G**	**p.D59E**	**Missense**	**VUS**	**PM2_P, PP3**	**Juvenile**	**7.0**	**1**	**Morocco**
c.214G > T	p.E72*	LoF	P	PM2_P, PVS1_VS, PM2_M, PS3_P	Late-infantile		1	Lebanon
**c.224del**	p.P75Hfs*41	**LoF**	**LP**	**PVS1, PM2_P**	**Late-infantile**		**1**	**Iran**
**c.244G > C **^**‡**^	**p.G82R**	**Missense**	**VUS**	**PM2_P, PP3, PP1_P**	**Late-infantile**		**1**	**Mexico**
**c.247G > A**	**p.D83N**	**Missense**	**VUS**	**PM2_P, PP3, PM3_P**	**Late-infantile**	**4.7**	**2 (1)**	**Egypt**
**c.248A > G**	**p.D83G**	**Missense**	**VUS**	**PM2_P, PM3_P**	**Late-infantile**		**1**	**Saudi Arabia**
**c.257A > G**	**p.H86R**	**Missense**	**VUS**	**PM3, PP3, PM2_P**	**Late-infantile**	**3.0, 4.0**	**5**	**Egypt**
**c.281C > T**	**p.P94L**	**Missense**	**VUS**	**PM2_P**	**Late-infantile**		**1**	**Egypt**
c.299 T > G	p.L100R	Missense	VUS	PM2_P, PP3, PM3_P	Late-infantile		1	Egypt
c.308G > A ^§^	p.R103Q	Missense	LP	PM5, PM3, PM2_P, PP1, PP3	Adult		1	Colombia
c.316dup	p.R106Pfs*26	LoF	P	PVS1_VS, PM2P_P, PM3	Late-infantile	3.1, 5.1	2	Pakistan, Saudi Arabia
**c.350 T > G**	**p.I117S**	**Missense**	**VUS**	**PM2_P, PP3, PM3_P**	**Adult**		**1**	**Lebanon**
**c.379C > T**	**p.H127Y**	**Missense**	**VUS**	**PM2_P, PP3, PM3_P**	**Late-infantile**	**5.5**	**1**	**Turkey**
c.395_396del	p.S132Cfs*18	LoF	P	PVS1_VS, PM3, PM2_P	Late-infantile	3.0, 3.1, 4.0, 4.1	6 (1)	Saudi Arabia (3), Egypt (2), Turkey
**c.397_398del**	p.V133Qfs*17	**LoF**	**P**	**PVS1_VS, PM2_P, PM3**	**Late-infantile**	**0.1**	**2**	**Iran**
c.406C > T	p.R136C	Missense	LP	PM2_P, PP3, PM3, PP1_P, PM5	Late-infantile	3.5	1	Egypt
c.407G > A	p.R136H	Missense	LP	PM2_P, PP3, PM3_S, PM5	Late-infantile	3.4	2	Tunisia, Libya
**c.434A > C**	**p.H145P**	**Missense**	**VUS**	**PM2_P, PP3**	**Late-infantile**		**1**	**Egypt**
c.434A > G	p.H145R	Missense	VUS	PM2_P, PP3	Late-infantile		1	Saudi Arabia
c.445C > T	p.R149C	Missense	VUS	PM2_P, PP3, PM5, PM3_P	Late-infantile		1	Egypt
c.461_463del	p.I154del	In-frame	P	PM4, PM2_P, PS3_P, PM3_S, PP1_M	Late-infantile		4	Brazil (2), Turkey, Algeria
c.476C > T	p.P159L	Missense	VUS	PM2_P, PP3, PM3	Late-infantile	2.1, 3.0	3	Iran (2), Turkey
**c.486 + 2 T > A**	**p.?**	**LoF**	**LP**	**PVS1_S, PM2_P, PM3_P**	**Late-infantile**	**6.1**	**1**	**Egypt**
**c.486 + 2 T > C**	**p.?**	**LoF**	**LP**	**PVS1_S, PM2_P, PM3_P**	**Late-infantile**	**3.0**	**1**	**Tunisia**
**c.543-2A > G**	**p.?**	**LoF**	**LP**	**PVS1_S, PM2_P, PM3_P**	**Late-infantile**	**2.9**	**1**	**Egypt**
**c.552del**^**‡**^	p.F185Sfs*21	**LoF**	**LP**	**PVS1_VS, PM2_P**	**Late-infantile**		**1**	**Mexico**
**c.587G > T**	**p.C196F**	**Missense**	**VUS**	**PM2_P**	**Late-infantile**		**1**	**Iran**
c.659A > C	p.Y220S	Missense	VUS	PM2_P, PP3, PM3_M	Late-infantile		1	Iran
c.662A > C	p.Y221S	In-frame	VUS	PM3, PP1, PM2_P	Late-infantile	5.0	4 (1)	Lebanon (3), Pakistan
**c.715_717del**	**p.F239del**	**In-frame**	**VUS**	**PM2_P, PM4**	**Late-infantile**		**1**	**Egypt**
**c.757_759del**	**p.L253del**	**In-frame**	**VUS**	**PM2_P, PM3_P, PM4**	**Late-infantile**		**1**	**Egypt**
**c.765_785del**	**p.D256_L262del**	**Missense**	**VUS**	**PM4, PM2_P, PM3_P**	**Late-infantile**	**4.0**	**1**	**Pakistan**
c.768C > G	p.D256E	Missense	LP	PP1_S, PM2_P, PP3, PM3	Teenage	15	1	Pakistan
**c.773A > G**	**p.N258S**	**Missense**	**VUS**	**PM2_P, PP3, PM3_P, PP1_M**	**Late-infantile**	**4.0**	**3 (1)**	**Saudi Arabia**
c.775G > A^§^	p.G259S	In-frame	VUS	PM2_P, PP3, PM3_P	Adult		1	Colombia
c.794_796del	p.S265del	Missense	LP	PM2_P, PP1, PM3, PM4	Late-infantile	3.0, 3.0, 3.0, 3.5, 3.5, 4.0, 5.0, 5.6	30 (2)	Egypt (7), Iraq, Jordan (2), Kuwait (4), Lebanon (7), Saudi Arabia (9)
c.829_836delinsCCT^¶^	p.V277Pfs*5	LoF	P	PM2_P, PVS1_S, PM3_S	Late-infantile		1	Brazil

^†^Where data were available

^‡^Heterozygote c.244G > C/c.552del

^§^Heterozygote c.308G > A / c.775G > A

^¶^Heterozygote c.461_463del / c.829_836delinsCCT

**Fig. 2:**
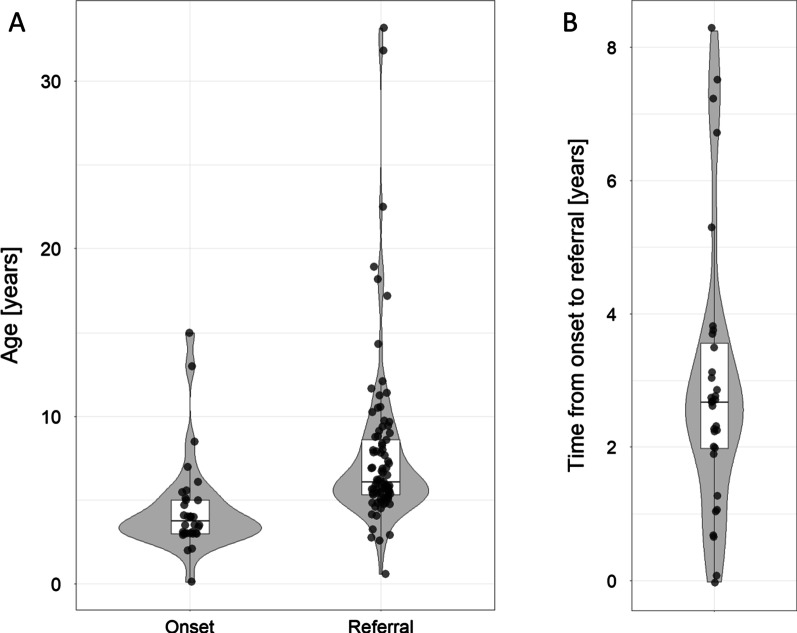
Patient age at disease onset and referral. Each dot represents one of 34 (**A**) or 32 patients (**B**) where sufficient information was available. Grey areas show the smoothed distribution of ages or years, white boxes the interquartile ranges, and lines inside each box the medians. **A** Ages at onset and referral. **B** Time from disease onset to referral for genetic testing for NCL disease

In addition, the ages of onset and at referral for genetic testing were provided for a subset of patients (Fig. 2A). The median age of onset was 3.8 years, with a range from less than a month to 15 years and an interquartile range (IQR) of 3.0–5.0 years (n = 34). The median age at referral was 6.1 years (range 7–33 years, IQR: 5.3–8.7 years, n = 88). The median time from disease onset to referral was 2.7 years and ranged from less than 1 month–8.3 years (IQR 2.0–3.6 years, n = 32) (Fig. 2B).

**Fig. 3:**
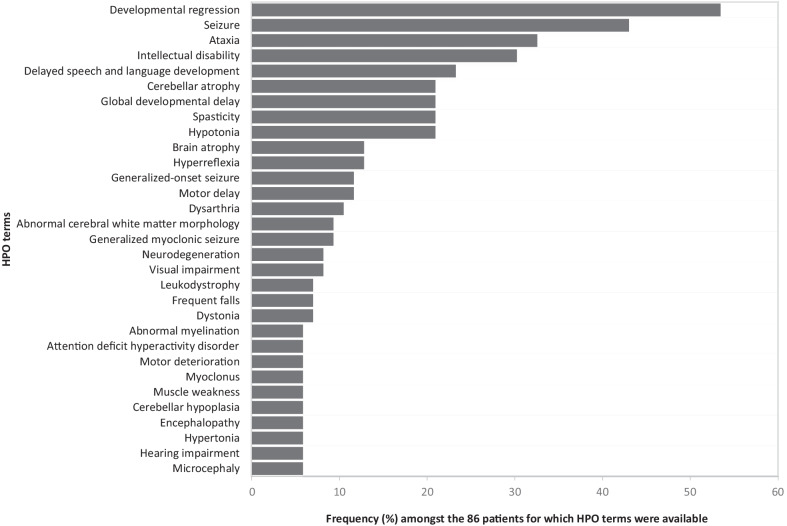
Clinical features and their frequencies in the cohort. HPO terms that were used in five or more patient diagnoses. Note terms with different levels of specificity are shown regardless of whether they might represent the same symptom. Consequently, data from many patients described in highly specific but rarely used terms could not be included

Clinical symptoms were provided for 86 patients and catalogued into 213 Human Phenotype Ontology (HPO) terms. The most frequent were “Developmental regression” (n = 46, 53%), “Seizure” (n = 37, 43%), “Ataxia” (n = 28, 33%), and “Intellectual disability” (n = 26, 30%). Figure 3 shows the 31 terms that were used in five or more patients. Alternatively, grouping HPO terms at the “Phenotypic abnormality” level provided a low-resolution overview that revealed that after nervous and musculoskeletal systems, the eye was indeed the third most affected organ in CLN6 patients. The symptoms were diverse and included various degrees of visual impairment in 12 patients, macular and other retinal abnormalities in eight and abnormalities of eye movement in five.

### Age of onset and time to referral

**Fig. 4:**
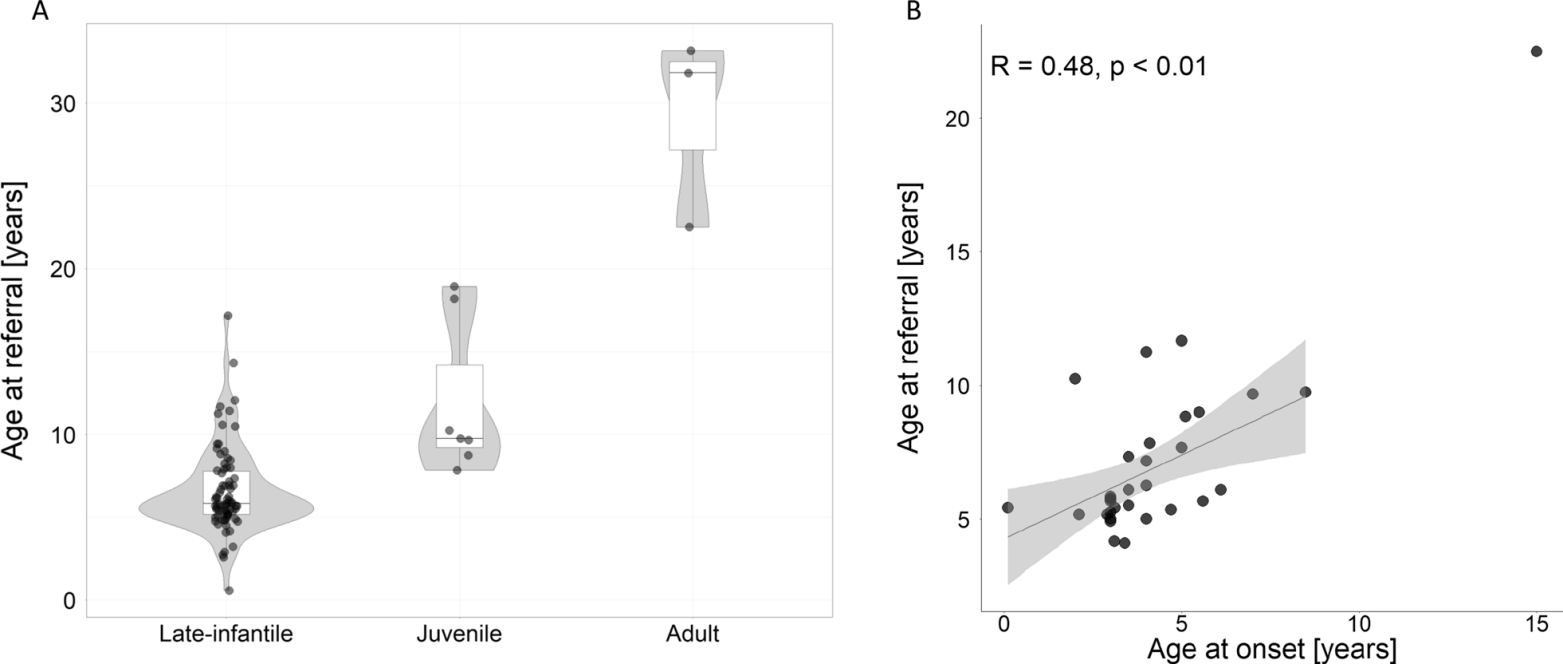
Association between age at onset and age at referral. **A** Age at referral for patients diagnosed with late-infantile, juvenile, and adult-onset disease. The adult-onset group comprised two cases of Kufs disease type A, and one case of teenage progressive myoclonic epilepsy. Each dot represents one of 88 patients for which age was provided. Grey areas show the smoothed distribution of ages or years, white boxes interquartile ranges, and lines inside them the medians. **B** Correlation between age of onset and age of referral in 32 patients for which both ages were recorded. Also shown are a least square regression line with a 95% confidence interval (grey area), and the Pearson correlation coefficient (R) with significance level. A linear model was fitted without considering the outlier in the upper right corner of the plot

The ages at which patients were referred for genetic testing differed according to disease onset type as would be expected (Fig. 4A). The median referral age for patients with late-infantile.

CLN6 disease was 5.8 years, but with a wide range of from six months to 17.2 years, and IQR of 5.2–7.8 years. The median age at referral for juvenile-onset patients was 9.8 years (range 7.8–18.9 years, IQR 8.9–16.9 years). The age of referral for the three adult-onset patients was 22.5, 31.8, and 33.2 years. A weak correlation existed between the ages of onset and referral (Pearson R^2^ = 0.23, p < 0.01) when disregarding the adult-onset outlier (Fig. 4B).

### Known and novel *CLN6* transcript variants discovered in this study

**Fig. 5:**
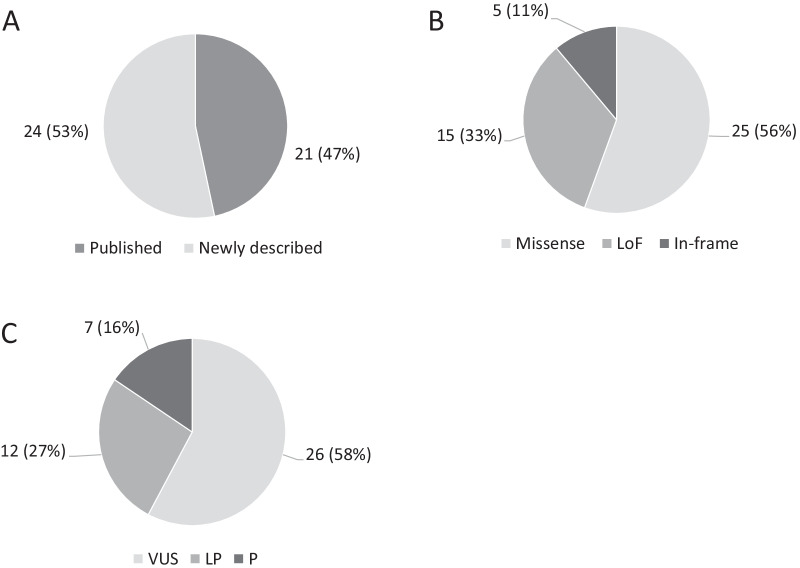
Characterization of the *CLN6* variant spectrum. Shown are the total numbers of distinct variants within each class in 94 homozygous mono-allelic, and 3 compound heterozygous bi-allelic cases (relative frequencies in brackets). **A** Distribution of distinct variants classified according to clinical significance as pathogenic (P), likely pathogenic (LP), or a variant of uncertain significance (VUS). **B** Distribution of the causative variants at the protein level. The loss of function (LoF) group comprises frameshift, splicing, nonsense, start loss mutations, and gross deletions or duplications. **C** Numbers of previously published vs. novel described variants in this study

Sequencing of the cohort identified 45 distinct *CLN6* transcript variants of which 24 (53%) have not been described previously (Fig. 5A). Frameshift, splicing, nonsense, start loss and gross deletions or duplications are genetic alterations with a high likelihood of causing complete loss of function (LoF) of a gene. This type accounted for 15 (33%) distinct variants, the remaining were 25 missense substitutions and five in-frame deletions (Fig. 5B). Among the novel mutations, 9 were LoF, 12 missense, and three in-frame. According to clinical significance, seven variants were classified as pathogenic (P), 12 as likely pathogenic (LP), and 26 as a variant of uncertain significance (VUS) (Fig. 5C). Novel variants comprised one P, seven LP, and 16 VUS.

**Fig. 6:**
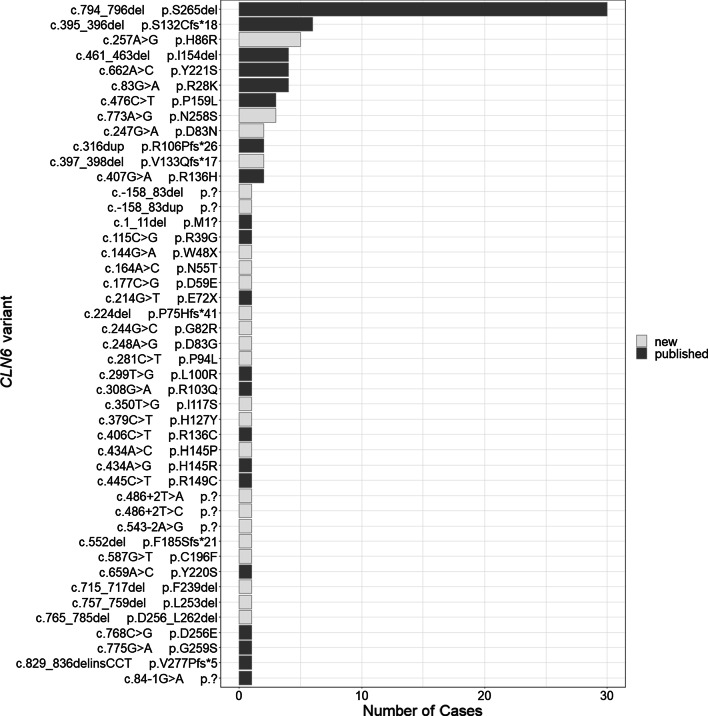
Frequency of individual *CLN6* variants among patient cases. All variants are shown, including those found in three compound heterozygous individuals. Lighter shaded bars indicate the number of cases with variants novel discovered by this study. See Table 1 for numbers and associated patient characteristics.

By far the most prevalent variant in our cohort was the in-frame deletion c.794_796del (p.265Sdel) which was present in 30 out of 97 patients (Fig. 6, further details in Table 1). The most frequent new variant, c.257A > G (p.H86R), was identified in five unrelated cases. Thirty-three variants were represented by a single case only.

### Association of *CLN6* variant classes with disease subtype and age of onset in homozygous cases

**Fig. 7:**
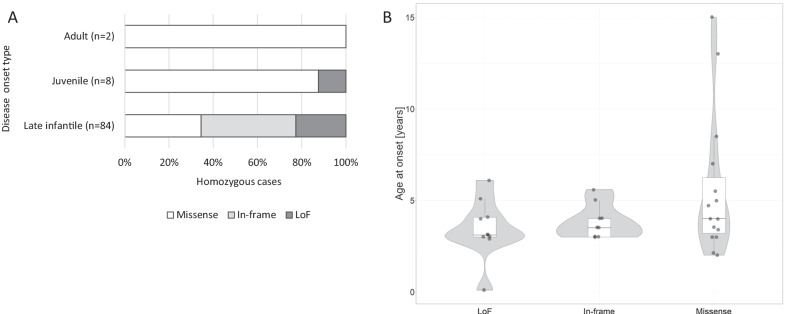
Association between age at onset and *CLN6* predicted coding effect types. **A** Alleles in each variant class, stratified by disease diagnosis. Shown are the percentages of distinct alleles in homozygote cases. The total numbers are shown in the y-axis labels for each disease type; no variant was found in more than one. White: missense, grey: in-frame, black: loss of function (LoF) mutations. **B** Age at onset in 34 cases grouped by *CLN6* variant type, plotted as in Figs. 2 and 4A. All patients in this dataset were homozygotes

There were only missense cases in the adult-onset and 87% of juvenile cases. (Fig. 7A). Moreover, the age of onset in patients homozygous for a missense variant displayed a wider range and slightly higher median than the ages of onset in the other two variant categories (Fig. 7B).

### Association of individual *CLN6* variants with disease subtype and age of onset

A detailed overview of the variants and the associated patient characteristics is given in Table 1. The most prevalent variant in the cohort, the in-frame deletion c.794_796del (p.S265del), was exclusively found in 30 (36%) (26 unrelated and 2 familial cases) homozygous patients with late-infantile onset. Likewise, none of the other alleles were shared by cases across disease onset groups. The missense variant c.83G > A (p.R28K) was exclusively present in three of the eight homozygous juvenile-onset patients (unrelated) and could be characteristic of this disease onset type. The four variants which were found only in adult-onset patients were c.768C > G, (p.D256E) and c.350 T > G (p.I117S) in homozygous, and c.308G > A (p.R103Q) and c.775G > A (p.G259S) as compound heterozygous genotype.

### Association of individual *CLN6* variants with geographical region

*CLN6* variant c.794 796del (p.S265del) was present in 22 out of 57 Middle Eastern, and in seven of the 31 North African patients from our cohort. Discounting sibling cases, the frequency was 39% and 21%, respectively. By contrast, this variant was absent in the nine patients from the Indian Subcontinent and Latin America. Some variants appeared to be more prevalent in specific countries. For example, all three unrelated patients from Oman, and one from Saudi Arabia had the c.83G > A (p.R28K) mutation. Of the novel described variants six originated from North Africa, four from the Middle East, one each from the Indian Subcontinent and Latin America. Cases with novel discovered variants were most prevalent in subjects from Egypt and Iran where they were present in over half of the cases (14/26 and 4 / 7, respectively). Specifically, c.257A > G(p.H86R) was found in five unrelated Egyptian individuals but none of the patients from other countries. New variants were much less frequent in Saudi Arabia (five of 21 cases) and Lebanon (one of 13 cases), the countries with the second and third-largest numbers of patients.

## Discussion

**Fig. 8:**
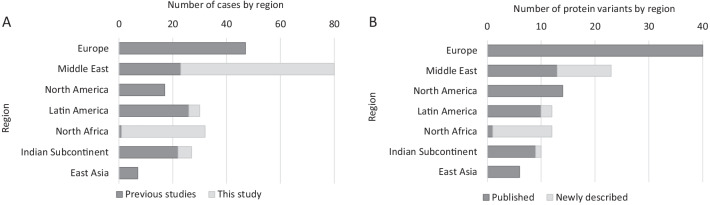
Size and representation of geographical regions in datasets. Dark grey fill: NCL Mutation and Patient Database (25), light grey fill: this study. Europe: Czech Republic, Greece, Ireland, Italy, Malta, Portugal, Spain, UK. Middle East: Arabic origin, Iran, Iraq, Israel, Jordan, Kuwait, Lebanon, Oman, Saudi Arabia, Turkey, United Arab Emirates. North America: Canada, USA. Latin America: Argentina, Brazil, Colombia, Costa Rica, Mexico, Venezuela. North Africa: Algeria, Egypt, Libya, Morocco, Sudan, Tunisia. Indian Subcontinent: Afghanistan, India, Pakistan, Sri Lanka. East Asia: China, Japan. **A** Patient numbers stratified by region. **B** Numbers of distinct variants reported for each region

We undertook a comprehensive analysis of the demographic, genetic, and clinical data collected from 97 diagnostic cases (85 unrelated and 6 pairs of siblings). To our knowledge, this is the largest cohort of CLN6 patients described so far for which genetic testing was performed at a single center. At the time of writing, the NCL Mutation and Patient Database [10, 25] listed 145 cases and 73 disease-associated CLN6 protein variants. We found 45 distinct variants, including 24 that were novel identified. Our report therefore significantly expands the published dataset. Of particular interest, we describe *CLN6* disease and variants which are prevalent in regions that were underrepresented in the previous dataset compared to Europe and North America (Fig. 8A, B). Causative variants of the *CLN6* gene have been described in a wide range of ethnic groups [26–28]. These studies suggested a regional predominance of certain variants such as c.214G > T (p.E72*) in Costa Rican patients [29, 30]. This variant was observed in our cohort of mainly Middle Eastern and North African patients only once. Instead, the late-infantile onset associated with c.794 796del (p.S265del) was predominant (Fig. 6). While our patients originated from twenty different countries, by far the highest numbers were from Egypt (27%), Saudi Arabia (22%), and Lebanon (13%) (Table 1). Egypt was also the country with the highest number of novel discovered alleles, which represented more than half of the distinct variants in this population. Interestingly, the c.257A > G (p.H86R) variant was found in five out of 23 unrelated Egyptian individuals but not in patients from any other countries. Together, these data confirm the genetic and ethnic heterogeneity of CLN6 disease [10]. They also highlight a need that has been motivating the CentoMD® database: Better characterization of rare diseases in populations that are currently underrepresented in public data, to improve patient care, and to generate novel insights into genetics and disease mechanisms [23].

Percentages of missense, in-frame, and LoF mutations were similar in novel and previously described variants. A limitation of most studies, including ours, is that the sequencing method can fail to detect LoF due to large-scale genomic alterations: an example is a rare 12 kb deletion involving exon 1 of the *CLN6* gene recently described in two Japanese patients [31]. One of the new variants could be classified as pathogenic due to being an amino acid sequence deletion of considerable size.

Among the 31 cases in our cohort with novel discovered variants 91% were late-infantile, 6% juvenile, and 3% adult onset / Kufs type A. Ignoring additional disease subtypes and missing information, the NCL Mutation and Patient Database [10] lists 110 cases with these clinical descriptions. Of these, 85% were late-infantile, 3% juvenile, and 13% adult onset / Kufs type A or B. Therefore, the distribution of onset types in cases with the novel discovered variants were similar to the published frequencies. The correlation between the ages of onset and referral was weak, and the time from first symptoms to referral for genetic diagnosis ranged from less than a month to up to eight years (Fig. 4).

Symptoms involving the nervous system, the musculoskeletal system, and the eye were the most common. In agreement with the current literature, we found no significant gender disparities in our cohort for patient numbers, age of onset, and occurrence of clinical symptoms (not shown). An interesting question is how the described variants can help to predict the onset and clinical symptoms of the disease. Our preliminary results suggest a trend for missense variants to be associated with a later onset (Fig. 7) but a detailed analysis was beyond the scope of this study. However, we hope that by increasing knowledge of the mutational spectrum and raising awareness of the disease this study will contribute to earlier diagnosis for CLN6 patients worldwide. Early and accurate identification of the genetic cause will be critical for effective treatment, including the gene therapy approaches as have been recently started [21] for this devastating progressive disease.

## Conclusions

We report the largest single-center cohort of CLN6 patients analyzed so far. It considerably expands the public data on CLN6 disease and *CLN6* mutational spectrum, especially for North Africa and the Middle East. It is hoped that this study will raise awareness for CLN6 disease and reduce the time from first symptoms to diagnosis for patients and their relatives worldwide. Including *CLN6* in the genetic diagnosis is recommended for individuals presenting with developmental regression, seizures, ataxia, intellectual disability, and ocular symptoms.

## Methods

### Patients and study design

A retrospective cross-sectional study was performed to investigate the clinical and mutational spectrum of CLN6 disease. It involved 97 subjects submitted for routine genetic diagnosis of CLN genes between January 2010 and October 2020 at Centogene GmbH (Rostock, Germany). Clinical symptoms were the cause of referral in 72 cases, for the rest no information was provided.

### Sample preparation and genetic analysis

All procedures were undertaken according to the provisions of the German Gene Diagnostic Act (Gendiagnostikgesetz) and the General Data Protection Act (Bundesdatenschutzgesetz) to guarantee confidentiality and data protection. The samples were processed at Centogene GmbH (Rostock, Germany) in a facility certified under the Clinical Laboratory Improvement Amendments of 1988 (CLIA), accredited by the College of American Pathologists (CAP). Samples were provided either as extracted DNA, EDTA blood, dried blood spots (DBS) on filter cards (CentoCard®, Centogene GmbH, Rostock, Germany), amniotic fluid, or saliva. DNA extraction was done on a QIAsymphony instrument using reagents and kits recommended by the manufacturer (Qiagen, Hilden, Germany). Procedures used by us for variant screening have been described previously [23, 32]. Depending on the referring physician´s request, sequencing was performed either as whole-exome sequencing (WES), as gene panel in the CentoMetabolic® or Ceroid lipofuscinosis panels or for CLN6 alone. For gene panel sequencing, a custom double-stranded DNA capture bait pool was used to selectively enrich the coding regions, including 10 bp of flanking intronic sequences and known relevant variants beyond the coding regions, based on HGMD® and an in-house databank. Libraries were generated with Illumina compatible adaptors and sequenced on an Illumina platform (Illumina, San Diego, CA) to obtain ≥ 20 × coverage depth for > 99.5% of the targeted bases. Missing fragments were completed by Sanger sequencing when necessary. For WES, human consensus coding sequences were enriched from fragmented genomic DNA using the Nextera Rapid Capture Exome kit (Illumina) / SureSelect Human All Exon V6(Agilent)/ TWIST Human Core Exome (Twist Bioscience). The generated libraries were sequenced on an Illumina platform to an average coverage depth 70–100 ×. Any relevant variants detected by WES were validated by Sanger sequencing in both directions.

An in-house bioinformatics pipeline was applied for read alignment to the GRCh37/hg19 genome assembly, variant calling, annotation, and comprehensive variant filtering. The investigation focused on coding exons and flanking ± 10 intronic bases. Results were reviewed, interpreted, and reported by our scientific and medical experts. All potential disease-causing variants, including those reported in HGMD®, ClinVar, and in our databank were considered. Detected variants were classified according to published ACMG guidelines as pathogenic (P), likely pathogenic (LP), and variant of unknown significance (VUS) [33–35]. Clinical data provided by the referring physician were annotated in conformity with the Human Phenotype Ontology (HPO) nomenclature [36].

### Statistical analysis

Medians, median-unbiased quartile ranges, and the correlation coefficient (Pearson´s R) and its significance were calculated using the stats package in R.

## Data Availability

Data on individual samples and research participants are not publicly available because of data privacy.
